# Inclusion of a computerized test in ADHD diagnosis process can improve trust in the specialists’ decision and elevate adherence levels

**DOI:** 10.1038/s41598-024-54834-y

**Published:** 2024-02-22

**Authors:** Ephraim S. Grossman, Itai Berger

**Affiliations:** 1 Department of Education, Ariel University, Ariel, Israel; 2 Pediatric Neurology, Pediatric Division, Faculty of Health Sciences, Assuta Ashdod University Medical Center, Ben-Gurion University of the Negev, Beer-Sheva, Israel; 3 The Paul Baerwald School of Social Work and Social Welfare, The Hebrew University of Jerusalem, Jerusalem, Israel

**Keywords:** Psychology, Health care

## Abstract

Attention deficit and hyperactivity disorder (ADHD) affects many life aspects of children and adults. Accurate identification, diagnosis and treatment of ADHD can facilitate better care. However, ADHD diagnosis and treatment methods are subject of controversy. Objective measures can elevate trust in specialist’s decision and treatment adherence. In this observational study we asked whether knowing that a computerized test was included in ADHD diagnosis process results in more trust and intention to adhere with treatment recommendations. Questionnaires were administered to 459 people, 196 men, average age = 40.57 (8.90). Questions regarding expected trust and adherence, trust trait, trust in physician and health-care-institutions, and ADHD scales followed a scenario about parents referred to a neurologist for sons’ ADHD diagnosis. The scenario presented to the test group (n = 185) mentioned that a computerized test was part of the diagnostic process. The control group scenario didn’t mention any computerized test in the diagnostic process. Test group participants expressed more trust in the diagnosis and greater levels of intention for treatment adherence. Group differences in intention for treatment adherence were mediated by trust in decision. Inclusion of a computerized test in ADHD diagnosis process can improve trust in the specialists’ decision and elevate adherence levels.

## Introduction

In health care settings, trust and communication serve (among other virtues) as a tool for better patient care and patient satisfaction. Effective and efficient communication is a predictor of medical practice and delivery of health care services^1^. Although evidence shows that in most cases patients continue to trust physicians to act on their best interests, there is growing concern that the rapid changes in health care systems and social media implications placed a great pressure on that trust^2^. In recent years more patients seek medical advice and personal recommendations in sometimes untrustworthy and in some way dangerous surroundings such as random internet sites^3^. A growing number of patients are recording the clinical encounters^4^. At the same time technologies are developed in order to aid the diagnostic processes and it is important to study the influence of such tests on patients trust in the diagnosis^5^.

This trend is especially prominent in Attention Deficit and Hyperactivity Disorder (ADHD)^6^. ADHD is considered one of the most common neurobehavioral disorders of childhood and among the most prevalent chronic health conditions affecting school-age children^7^. However, the disorder is the subject of much controversy regarding diagnosis and treatment methods, both in the medical literature and the public media^8^.

ADHD is a chronic neurodevelopmental disorder affecting most life aspects of young children including academic^9^, social^10^ and home^11^ domains. In recent years, studies confirm that this disorder accompanies many people into adulthood^12^. When ADHD effects persist into adulthood, they are associated with higher mental co-morbidity, mood disorders, educational and interpersonal impairments^13^. Young people who were diagnosed as having ADHD in childhood tend to have lower levels of educational and occupational status compared to people without ADHD^14^. ADHD is also associated with higher mortality rates, due to suicide, unintentional injury, and homicide^15^.‏ Accurate identification, diagnosis and treatment of ADHD can lead to better care of these patients^16–18^, thereby preventing or easing many long-term implications^19^. Since children, adolescents and adults with ADHD experience functional impairment and poor health-related quality of life, the efficacy of appropriate ADHD interventions, medication, social and psychological support, extends beyond symptom control and may help reduce the related but distinct impairments and deficits in these patients^20^. However, since interventions can end up, in some cases, with mixed outcomes^21^ a trustful caregiver should be available to explain such results to patients and families.

### ADHD diagnosis

ADHD diagnosis is based on clinical evaluation^22^ using reports obtained from at least two sources and ruling out other possible reasons. In the case of children, the diagnosis is mostly based on parental and educational staff. As systematic as these reports can be they are affected by subjective interpretations^23^. Social and personal perceptions can influence behavioral expectations and perspectives^24^ which can add to the subjectivity of the reports. Low rates of agreement between parents’ and teachers’ reports are thus frequently observed^25^ Integration of multiple sources of information is essential in the face of such discrepancies^24^ in order to come up with a valid diagnostic decision which is crucial for optimal treatment. Closely following is the need to make the patients, or parents, trust the physicians’ conclusions.

### Objective diagnostic tools

One possible way to improve the accuracy of diagnosis, ADHD diagnosis in particular, is to use objective measures as part of the diagnosis process. Brain activity and computerized neurocognitive tests were considered as candidates for this task^23^ as it can allow accuracy and consistency to the process^26^ especially in adults beyond the brain development stage. Other attempts for utilizing known physiological differences between those diagnosed with ADHD and controls as reliable and ready-to-use biomarkers for ADHD diagnosis did not^27^ meet the criteria defined by the world federation of ADHD task force^28^. However, adding an objective behavioral measure to the diagnostic process is viewed by some as a game changer^29^. Knowledge about results obtained by an objective computerized test made clinicians more confident in the diagnosis and helped them arrive at decisions faster^30^.

A second advantage that can be achieved by using objective computerized tests, dependent or independent of the first issue, is the possibility to elevate patients trust in the result of the diagnosis and improve treatment adherence^27^.

As technology advances and computerized tests become available and accurate diagnostic tools (for example see^31^) their use will probably be meaningful both for the clinician and patient who will be able to rely on computerized test result. According to Pezzo, Nash, Vieux, & Foster-Grammer^32^, in some cases, considering the results of a computerized test may protect the physician. Studies show that computerized tests can correctly identify children with and without ADHD^7,33^. At this point making use of an additional valid objective tool alongside to the diagnostic process may be considered. In the current study, we looked for differences in parental trust of physicians’ diagnosis of a hypothetical referral of a child for ADHD diagnosis with or without knowledge about results of a computerized test.

### Patient trust

The level of trust a patient has in the physician has important implications on the course of diagnosis and treatment. Patients trust in their doctors is associated with satisfaction and treatment adherence^34^, positive patient health outcomes (see^35^ for review) and reduces health care costs^36^. Similar results emerged in a recent meta-analysis^37^ which found that a large body of studies show that patients reported to be more satisfied with treatment, to show more beneficial health behaviors, less symptoms and higher quality of life when they had higher trust in their health care professional. On the other hand, a lack of patient—physician or patient—healthcare system trust has been associated with negative outcomes. Poorer health status, decreasing adherence to medication treatment, and a tendency to ignore suggestions for lifestyle modification^38^ and in some cases more distress^39^. Trust may be especially important in the case of ADHD diagnosis, a process in which the physician interprets subjective reports^23^ advocated by parties (patient / parents / educators) which may not fully agree each with the other. Furthermore, many parents have an initial negative view of medical treatment for ADHD which is perceived as a social, emotional, or psychological problem^40^. They are suspicious and fearful about it and eventually state that they considered the neurologist’s explanation as the single main factor affecting their change to positive attitudes^6^.

We propose that increasing the trust in the diagnosis can influence the adherence to treatment thereby improving personal outcomes (not in the scope of this research). The importance of early intervention, both for each patient and for the society, led the purpose of the current study to seek possible mechanism for enhancing the effectiveness of treating ADHD. We hypothesize that the use of an objective measure as part of the diagnostic process can be related to treatment acceptance and adherence. The aim of the present study was to evaluate in a method of questionnaire-based observational study, if using a computerized test in the diagnostic process for ADHD improves the trust in the diagnosis and the intention to adhere with recommended treatment.

## Methods

This is a questionnaire-based observational study.

### Participants

Questionnaires were distributed by links sent over social media or by social science students who volunteered to distribute paper questionnaires. All returned data was anonymously coded. Out of the 492 questionnaires returned, 33 were erased due to not signing the informed consent paragraph or missing important data or not fulfilling study inclusion requirements. Participants were supposed to be 25 years old or over and have one child or more. This was important so participants could relate to the situation described in the scenario presented. Participants over the age of 60 were excluded as well. The final sample included 459 people, 196 men and 245 women (18 did not report their gender), average age was 40.57 (SD = 8.90). All are parents to children. Fifty-six (12%) gave a positive response for suspecting themselves as having ADHD and 122 (27%) said yes in response to suspecting that any of their children had ADHD.

### Variable measures

The questionnaire administered to participants included basic demographic information, two questions about being diagnosed as having ADHD or having a child diagnosed as having ADHD. A case scenario created for the current study about parents referred to a neurologist with their son for ADHD diagnosis. The scenario was followed by four questions regarding the expected trust and adherence the parents will have of the neurologists’ diagnosis and treatment decision. Following were a trust scale, a trust in physician and in health care institutions questionnaire, and a self-report of attention deficit scale. The reliability of these variables were alpha = 0.78 for trust in diagnosis (2 items) and alpha = 0.86 for adherence (2 items).

#### Manipulation

Participants were asked to answer the different questionnaires after reading a scenario, about parents receiving complaints about their son’s learning and behavior and being referred to a specialist. The parents take the child to see a neurologist where they present information and questionnaires brought from school and from their home perspective. The neurologist then conducts a basic functions examination and summarizes his impression and suggested treatment based on the various sources of information including the results of a computerized diagnostic test. The full scenario (translated into English) appears in appendix 1. The words written in the scenario, presented in appendix 1 in bold, were part of the case story presented to the test group but were not included in the case presented to the control group. In other words, the words in bold compose the manipulation.

The trust scale was adopted from Evans and Revelle’s Survey and Behavioral Measurements of Interpersonal Trust^41^. The original scale has 21 items, 10 about trustworthiness and 11 about trust-in-other-people, coded on a Likert scale between “Does not describe me at all” (1) and “Accurately describes me” (5). In the original study only 7 of the items with face validity for trust loaded on the trust scale (alpha = 0.73). For the current study all 11 trust-in other-people questions were administered. Calculating the trust score with either 11 or 7 items yielded the same reliability score (alpha = 0.77) and the same pattern of results on the statistical analysis. The 11 items possibility is used hereafter.

The Multidimensional Trust in Health-Care Systems Scale^42^ has 3 subscales scored on a 5-point Likert scale with scores ranging from 5 (strongly agree) to 1 (strongly disagree). Item 4 and item 15 are reverse scored. Summary scores consisting of the average of the individual items were created such that higher subscale scores represent greater trust in healthcare systems. For the present study only the trust-in-health-care-providers (10 items, alpha = 0.85) and trust-in-health-care-institutions (3 items) subscales were used.

Self-assessment of ADHD was measured by the DuPaul questionnaire^17^ which includes 18 items depicting DSM-5 ADHD symptoms. The DuPaul questionnaire is regularly used for parent and teacher assessment of child ADHD. Responses are on a 0–3 scale. The sum of scores each subject endorsed was calculated, alpha = 0.91.

### Procedure

All methods were performed in accordance with the relevant guidelines and regulations. Paper and online questionnaires were administered to acquaintances and through social media according to the study design approved by the Ethics Committee for research involving human subjects of the Faculty of Social Sciences and Humanities at Ariel University. For printed questionnaires each student randomly received one type (scenario which includes / does not include a mention of a computerized test as part of the diagnostic process) of questionnaire to distribute. However, neither the distributers of the questionnaires nor the participants were informed that there were two slightly different scenarios. Participants were asked to read the scenario and reply to the questionnaire after being updated that the study was about attitudes towards ADHD diagnosis. Informed consent was obtained from all participants.

### Statistics

All of the data collected for the study was saved for statistical analysis in a SPSS version 29 file. Power analyses for detecting a medium effect size (0.5) comparing independent means and for a regression with 10 predictors (0.15) required a maximum sample size of 210. The current sample of 459 was thus sufficient for the study. All analyses were two-tailed with significance level set to 0.05. First the scores of the study dependent variables were calculated by averaging or summing over the items of each questionnaire. Next, an independent samples t-test was used to find differences in the dependent variables between the study groups. Confidence intervals of differences and Cohen’s d statistics for effect size were added when differences were found. A linear regression with 3 steps was used to understand the unique contribution of the different variables to the intention to adhere to treatment recommendations while controlling in each step for the variables entered in the previous steps. First, we entered demographic variables, followed by personal variables as suspecting oneself and children as having ADHD, ADHD scores, personal traits of trust and healthcare related trust. In order to learn about the specific contribution of the study manipulation and acquired trust in the diagnosis we added in the last two steps the group variable and the specific trust in diagnosis.

Finally, In order to emphasize the mediational role of the acquired trust in the diagnosis on the relation between knowing that a computerized test was part of the diagnosis and intention to adhere with treatment recommendations, we used the model number 4 PROCESS macro in SPSS^43^, which calculates a regression analyses. This macro assesses the magnitude of the indirect effect of the predictor on the outcome through the mediator while considering gender, age, suspecting oneself has ADHD, suspecting any of ones’ children has ADHD, ADHD score, general trust, trust in health care provider and trust in health-care-institutions as covariates.

## Results

Independent samples t-tests confirmed that the average age of study (mean = 40.89, SD = 9.10) and control groups (mean = 40.36, SD = 8.77) did not differ (*t*_(457)_ = 0.62, *P* > 0.05) nor was there a significant relation between gender proportions in study (Male: 50%; Female: 50%) or control (Male: 41%; Female: 59%) groups (χ^2^_(1)_ = 3.66, *P* > 0.05). Similarly, the distribution of family status was independent of being part of study or control groups (Currently married: 88% vs 90% in study and control groups respectively, (χ^2^_(1)_ = 0.55, *P* > 0.05). The averages (SD) of the outcome variables for the two groups are presented in Table 1.

**Table 1:** Average scores (SD) of the variables for the two study groups.

	Computerizes test results **not** mentioned in scenario *(n* = 275)		Computerizes test results mentioned in scenario *(n* = 189)		Group differences
	% / Mean	SD	% / Mean	SD	
ADHD (self)	Yes-10% No-90%		Yes-15% No-85%		χ^2^_(1)_ = 2.40, NS
ADHD (child)	Yes-30% No-70%		Yes-22% No-78%		χ^2^_(1)_ = 2.99, NS
ADHD score	15.53	(10.43)	14.21	(9.37)	*t*_(457)_ = 1.38 NS, CI = − 3.19–0.56
Trust trait	3.85	(0.56)	3.92	(0.54)	*t*_(457)_ = 1.30 NS, CI = − 0.35–0.52
Trust in health provider	3.43	(0.82)	3.35	(0.76)	*t*_(456)_ = 1.17 NS, CI = − 0.24–0.06
Trust in health Institutions	2.73	(0.69)	2.77	(0.71)	*t*_(454)_ = 0.66 NS, CI = − 0.09–0.18
Trust in diagnosis	65.05	(19.10)	71.97	(17.00)	*t*_(457)_ = 3.98, *p* < 0.01, CI = − 3.50–10.34, Cohen’s *d* = 0.38
Adherence intention	67.63	(25.39)	72.49	(24.16)	*t*_(457)_ = 2.05, *p* < 0.05, CI = 0.20–9.22, Cohen’s *d* = 0.19

Values of binary variables: ADHD (self) and ADHD (child): 0 = no, 1 = yes; Group: 0 = control group, 1 = study group.

ADHD / Trust trait / Trust in Health provider / Trust in Health Institutions are continuous variables.

Our hypothesis concerning significantly higher expression of trust in the diagnostic decision and more intention to adhere to the suggested treatment among subjects who received the scenario which mentioned the computerized test was initially confirmed by an independent samples t-test, as presented in Table 1. The differences between the groups were not significant for all other trust variables (all *P*’s > 0.05).

Pearson correlations confirmed that the three trust scales were significantly correlated with the expected parental reactions to the scenarios. The mediating trust in diagnosis variable was positively correlated with the trust trait (*r*_(459)_ = 0.13, *p* < 0.01), trust in health provider (*r*_(458)_ = 0.26, *p* < 0.001), and trust in health Institutions (*r*_(456)_ = 0.25, *p* < 0.001). The expected adherence variable was positively correlated with the trust trait (*r*_(459)_ = 0.12, *p* = 0.014), trust in health provider (*r*_(458)_ = 0.17, *p* < 0.001), and trust in health Institutions (*r*_(456)_ = 0.17, *p* < 0.001). Correlations between participants ADHD scores and expected reactions to the scenarios were not significant: trust in diagnosis Χ ADHD (*r*_(459)_ = − 0.02, *p* > 0.05), expected adherence Χ ADHD (*r*_(459)_ = − 0.08, *p* > 0.05). The correlation between trust in diagnosis and expected adherence was significant (*r*_(459)_ = 0.62, *p* < 0.001).

Following these results and known relations between adherence and gender, age, ADHD severity and family history^40,44^ we performed a regression analysis which confirmed that intention to comply was related to levels of trust in the diagnostic decision on the final step more than to any other variable. Table 2 presents the results of a linear regression with 3 steps. On the first step trust trait showed a tendency for being related with expected adherence while trust in health institutions was found to significantly contribute to expected adherence. On the 3rd step ‘trust in diagnosis’ becomes the only variable significantly related to expected adherence to treatment recommendations. Of importance is the fact that it replaces the significant trust in health institutions variable and more importantly the group effect obtained on the 2nd step.

**Table 2:** Linear regression for predicting diagnosis acceptance (*n* = 434).

Variable	ΔR^2^	*B*	95% CI for *B*		*SE B*	β	*T*	*p*
			*LL*	*UL*				
Step 1	.031**							
Gender		− 0.48	− 5.47	3.96	2.40	− 0.02	− 0.20	.841
Age		0.07	− 0.20	0.34	0.14	0.03	0.51	.611
ADHD (self)		− 0.04	− 9.62	1.39	3.89	− 0.01	− 0.01	.992
ADHD (child)		0.04	− 2.89	7.40	2.89	0.01	0.01	.989
ADHD score		0.06	− .015	0.38	0.13	0.03	0.48	.634
Trust trait		4.53	0.18	9.54	2.39	0.10	1.89	.058
Trust in Health Provider		2.62	− 0.76	5.91	1.69	0.08	1.55	.122
Trust in Health Institutions		4.97	0.96	8.44	1.90	0.14	2.62	.009
Step 2	.007*							
Gender		− 0.11	− 5.05	4.37	2.40	− 0.00	− 0.05	.964
Age		0.06	− .021	0.33	0.14	0.02	0.39	.697
ADHD (self)		− 0.96	− 10.02	0.96	3.90	− 0.01	0.25	.806
ADHD (child)		0.62	− 2.50	7.78	2.89	0.01	0.21	.832
ADHD score		0.08	− 0.13	0.39	0.13	0.03	0.63	.529
Trust trait		4.40	0.05	9.38	2.38	0.10	1.85	.065
Trust in Health Provider		2.88	− 0.47	6.20	1.70	0.09	1.71	.089
Trust in Health Institutions		4.77	0.75	8.21	1.90	0.13	2.51	.012
Group		5.03	0.47	10.04	2.46	0.10	2.05	.041
Step 3	.343***							
Gender		0.46	− 3.62	3.94	1.93	0.01	0.24	.810
Age		0.02	− 0.18	0.26	0.11	0.01	0.16	.885
ADHD (self)		2.92	− 8.62	0.19	3.14	− 0.04	0.93	.353
ADHD (child)		− 0.13	− 3.75	4.51	2.32	− 0.00	− 0.06	.954
ADHD score		0.10	− 0.01	0.41	0.11	0.04	0.93	.355
Trust trait		1.59	− 1.77	5.75	1.92	0.03	0.83	.407
Trust in Health Provider		− 0.52	− 3.47	1.95	1.37	− 0.02	− 0.38	.704
Trust in Health Institutions		0.66	− 2.68	3.40	1.55	0.02	0.43	.671
Group		–1.38	− 4.88	2.96	2.02	− 0.03	− 0.68	.494
Trust in diagnosis		0.85	0.74	.096	0.06	0.63	15.36	< .001
Total R^2^	.385***							

Statistics of 3 steps of a linear regression predicting intended adherence by trust in diagnosis over personal variables, trust variables and study grouping.

Values of binary variables: ADHD (self) and ADHD (child): 0 = no, 1 = yes; Group: 0 = control group, 1 = study group.

ADHD / Trust trait / Trust in Health provider / Trust in Health Institutions are continuous variables.

There was no evidence of multicollinearity as VIF values of all variables over the three steps were in the range of 1.00 < VIF < 1.33.

*P < .05; *** *P* < .001.

**Figure 1:**
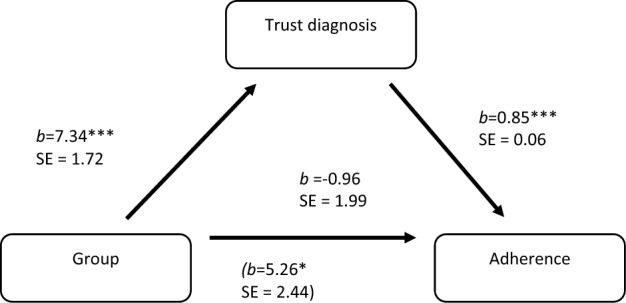
Mediation model of group > trust in diagnosis > adherence (*n* = 438). ‘Group’ values: 0 = control group (no computerized test mentioned), 1 = study group (computerized test mentioned in the scenario).

Next, we performed a mediation analysis with Hayes’ PROCESS computational procedure^43^ (model number 4), whereby group is associated with adherence via its indirect effect on trust in diagnosis. Gender, age, experience with ADHD in parent and child, ADHD score, and the three trust measures were included as covariates. First, there was a significant direct effect of group on adherence *b* = 5.26, *SE* = 2.44, *P* < 0.05. Second, there was a significant effect of group on trust *b* = 7.34, *SE* = 1.72, *P* < 0.001, Third, when the regression equation included both group and trust in diagnosis, only the latter predicted adherence, *b* = 0.85, SE = 0.06, *P* > 0.0001. For the summary of the mediation model of indirect effect of group manipulation on adherence intention the effect size was 6.21 (3.36–8.91), see Fig. 1. This suggests an increase of about 6% in intention to adhere due to knowing that a computerized attention test was involved in the diagnostic process that in turn increased trust in diagnosis.

## Discussion

We have found that people exposed to ADHD diagnostic procedure consisting of a computerized task expressed higher levels of acceptance of ADHD diagnosis and intention for adherence compared to people exposed to a similar scenario not including a mention of a computerized task. The study demonstrates that the existence of a computerized test as part of a child’s ADHD diagnosis procedure influence parents’ trust in the specialist’s decision over and beyond the contribution of a personal trait of trust or trust in the medical institutions. This finding is consistent with other recent studies which found high levels of acceptance and trust in the diagnosis when a computer-assisted and automated diagnostics tool was used (e.g.^5^).

Few factors may contribute to the difficulty of accepting ADHD diagnosis (for ones’ self or ones’ children). Apart of the emotional difficulty of accepting that ‘something is wrong’ with me/my child^45^, some features of this diagnostic structure may add to this difficulty. The lack of objective measures, lack of available biomarkers, and physiological tests for ADHD are probably part of the problem. Of the subjective tools, parental and educational staffs’ input are usually considered important factors, however, the agreement between these two sources of information is modest^46,47^ partly because parents and teachers base their impressions on different contexts^47^. The nature and salience of hyperactivity / impulsivity and attention may be the source of teachers’ and parents’ higher level of agreement for hyperactivity and impulsivity than for inattention^48^. The results of our study are in line with the conclusions of Hall et al.^49^ in suggesting that inclusion of an objective measure in the diagnosis process, supporting the decision made by the physician may contribute to greater acceptance of such diagnosis and to better adherence with a suggested treatment. The study of Hall et al. focused on the ability of objective measures to facilitate the diagnostic process by making it faster, more accurate, cost-saving and improve patient outcome. The current study emphasizes the psychological perspective whereby an objective measure can effect trust and indirectly facilitate treatment adherence. Such adherence may have better cost effectiveness, also improving patient outcome as previously seen^50^.

Previous studies showed that there is a significant although not consistent association between trust in the health care professional and health outcome^37^. Our marginal result concerning this variable may be related to this inconsistency. The current study suggests that inclusion of a “positive” result of a computerized test in addition to tests required by local guidelines can explain more of the variance of intention to adhere with the suggested treatment than any other trait we measured. This inclusion topped the potential effects of trust variables on expected treatment adherence.

The importance of a trustful relationship with the pediatrician was illustrated by Benin, Wisler-Scher, Colson, Shapiro & Holmboe^51^, who showed that trust or lack of trust was one of the main determinants of mothers’ decisions about vaccination. Benin et al. state that the reliance on trust is especially impressive, because of the special nature of vaccines, an issue in dispute and lack of mothers’ experience of relevant diseases. Similar results were recently obtained concerning COVID-19 vaccine intentions where peoples’ trust in their physician was strongly associated with vaccine likelihood^52^. Parent – physician relationship was found to be an important factor in child’s ADHD treatment decisions as well^53^. Here, our results demonstrate that acceptance and adherence to ADHD treatment may be affected by including an objective measure in the diagnostic process. This may be especially important in the case of ADHD where both diagnosis and medication treatment are under dispute. A careful balance is needed between human and computer intervention^54^ especially as one cannot impose on clients a test which is not part of the guidelines. However, in line with Coletti et al.^53^ and Brinkman et al.^55^, adding results of a computerized test to the explanation the physician gives the parents may result in better acceptance of medical choice of treatment.

The initial step of the regression shows the importance of the personal trait of trust and specifically trust in the medical system. This should not be surprising as people who “score higher” on the trust trait measure tend to reflect a greater tendency to adhere to the proposed treatment.

The results merit a separate consideration of the variables of trust related to physician and trust in health care Institutions. According to our analysis, when entered to the regression the trust in the physician was not related to expected adherence, while trust in health institutions did seem to be positively related to acceptance of the treatment recommendations. The lack of relation to trust in personal health provider in the current study does not contradict the above-mentioned importance of trust in physician. In our questionnaire the items about trust in physician led subjects to think about their relationship with their personal / family physician. However, for the scenario described in the current study this relationship was not relevant. In this case, the family physician, rather than being part of the diagnostic process, served as a technical referral to enable the parents receive treatment from the specialist. The Neurologist described in the scenario, may be perceived by the parents or by our participants, as part of the health care system or service. Thus, it is reasonable that the trust the participants feel towards the diagnostic process is related to their feeling towards the healthcare “system”.

It is important to keep in mind that the scenario presented to our participants asked about trust in a computerized test conducted in addition rather than instead of the guidelines for ADHD diagnosis. This raises the question of asking the clients, in this case parents of a child, to invest more time and money in the diagnostic process. Physicians should consider when to use and when not to use this additional tool. When it seems that the parents are hesitating but are willing to get another point of view an objective measure as provided by a computerized test may then be suggested. This may facilitate agreement and subsequently, as presented in the current data, trust and adherence.

### Limitations

This study is obviously a self-report study concerning an imaginary scenario. No actual testing of adherence or acceptance of a real diagnosis were tested. Report of an attitude to trust and to adhere with treatment in a theoretical scenario is not the same as having an actual feeling and attitude when personally involved. In addition, participants were not asked whether they or any of their children underwent an actual diagnosis process for ADHD. Such an experience may have had some influence on some of our respondents. However, the self-report of ADHD symptoms showed that the two groups were balanced regarding ADHD. ADHD is widely and commonly diagnosed, thus real-world situations where an objective measure, especially a computerized test is in use, must be tested for trust in diagnostic decision and adherence in comparison with cases where the additional tools are absent. However, we used a double-blind manipulation. Neither our research assistants nor the subjects were informed that two types of scenarios were presented. This gives some strength to the fact that people think that when a computerized test is part of the diagnostic procedure, they can trust the decision and adhere with treatment more than when the decision is based on subjective reports and impression of a specialist. At any stage of the regression analysis neither ADHD symptoms nor suspecting of having an attention problem or that one of one’s children has an attentional problem could predict adherence intention. This too may suggest that all participants related to the theoretical aspect of the scenario rather than letting any personal involvement influence their considerations.

## Conclusions

Inclusion of a computerized test in ADHD diagnosis process can improve trust in the specialists’ decision and can consequently elevate adherence levels. Studies testing this kind of placebo effect in real world situations are needed to further clarify this possibility.

### Supplementary Information

Supplementary Information.

## Data Availability

The datasets generated and analyzed during the current study are available on OSF data sharing at 10.17605/OSF.IO/927PE.

## Abbreviation

ADHD: Attention-deficit hyperactivity disorder
