# Supplementary material for: Inclusion of a computerized test in ADHD diagnosis process can improve trust in the specialists’ decision and elevate adherence levels
Source: Sci Rep. 2024 Feb 22;14:4392. doi: 10.1038/s41598-024-54834-y (PMC10884014; doi:10.1038/s41598-024-54834-y)
Supplement: Supplementary file 1 — Supplementary Information. [file 41598_2024_54834_MOESM1_ESM.pdf]

Appendix 1

**Inclusion of a Computerized Test in ADHD Diagnosis Process can Improve Trust in the Specialists' Decision and Elevate Adherence Levels**."

Ephraim S. Grossman[1] and, Itai Berger.

The scenario presented to study participants:

> About two months after the beginning of his third grade, Jacob's parents began to hear complaints about their son's learning and behavior. They were told that he does not listen to the teacher in class, does not open the textbooks on time, is late to return from the breaks and sometimes harasses his friends. Despite being a smart child, Jacob is unable to answer questions the teacher asks him during the lessons. The parents were referred to a neurologist and during their meeting told him about what they were hearing from school, about how they get along with Jacob at home and presented a questionnaire the teacher filled about Jacob. The parents were also asked to fill out a similar questionnaire. The neurologist conducted an easy examination of Jacob's basic functions and spoke to him briefly. The neurologist continued to talk to the parents **while Jacob conducted a computerized diagnosis test**. The neurologist then summarized everything and told the parents that according to his impression of whatever they and the educational staff described, on the basis of his examination and the questionnaires (Teachers' questionnaire 8/18, Parents' questionnaire 5/18) **and according to the computerized test score (-2.3)** he realizes that Jacob has Attention Deficit / Hyperactivity Disorder (ADHD) and should begin medical treatment.

After reading this scenario regarding Jacobs' parents the participants were asked the following four questions (Answers were rated on a 0 (not at all) -100 (To a great extent) scale):

1. To what extent do you think the parents should trust / rely on the neurologists' decision?
2. To what extent do you think the parents would trust / rely on the neurologists' decision?
3. To what extent do you think should the parents adhere to the medication treatment for Jacob?
4. To what extent do you think the parents will actually adhere to the medication treatment for Jacob?

Other study scales followed … (see methods)
